# A Borosilicide with Clathrate VIII Structure

**DOI:** 10.1021/jacs.2c04745

**Published:** 2022-07-25

**Authors:** Julia-Maria Hübner, Wilder Carrillo-Cabrera, Primoz Kozelj, Yurii Prots, Michael Baitinger, Ulrich Schwarz, Walter Jung

**Affiliations:** † Department of Chemistry, Centre for Analysis and Synthesis, Naturvetarvägen 14, 221 00 Lund, Sweden; ‡ Max-Planck-Institute for Chemical Physics for Solids, 01187 Dresden, Germany

## Abstract

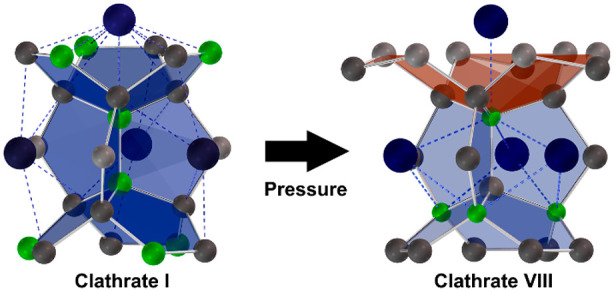

The high-pressure phase Na_8_B_*x*_Si_46–*x*_ (3 < *x* < 5) is the first representative of a borosilicide crystallizing in the rarely occurring clathrate VIII type structure. Crystals with composition Na_8_B_4_Si_42_ (space group *I*43̅*m*; *a* = 9.7187(2) Å; Pearson symbol *cI*54) were obtained at 5–8 GPa and 1200 K. The clathrate I modification exists for the same composition at lower pressure with a larger cell volume (*Pm*3̅*n*; *a* = 9. 977(2) Å; *cP*54). Profound structural adaptions allow for a higher density of the clathrate VIII type than clathrate I, opening up the perspective of obtaining clathrate VIII type compounds as high-pressure forms of clathrate I.

Intermetallic clathrates are framework compounds established by covalently bonded *p*- or *d*-elements enclosing filler atoms in polyhedral cages.^1^ Their significance is fueled by beneficial electrochemical properties,^2^ superconductivity,^3,4^ and an inherently low thermal conductivity, which qualifies them as potential thermoelectric energy materials.^5^ The broad variability of their chemical compositions opens a wide design space for tuning physical properties, e.g., from metallic to semiconducting behavior.^6^ The vast majority of clathrate representatives belong to type I (space group *Pm*3̅*n*), consisting of 20- and 24-atom cages (Figure 1a), or to the clathrate II structure (*Fd*3̅*m*) with 20- and 28-atom cages. The crystal structure of clathrate VIII^7^ (*I*4̅3*m*) offers (20 + 3)-atom cavities and is, thus, able to adapt to filler atoms that are too small to stabilize larger cages (Figure 1b). Representatives of this type are rare^8−13^ but of current interest, as they feature promising thermoelectric efficiency.^14,15^ The clathrate VIII pattern exhibits a higher density than the clathrate I arrangement,^9^ so it is reasonable to assume that type VIII is favored at high pressure. Herein, we will provide evidence that the clathrate VIII pattern is preferred at the cost of clathrate I upon enhanced compression in borosilicides Na_8_B_*y*_Si_46*–y*_ (3 ≤ *y* ≤ 5). Single crystals of the clathrate I and VIII modifications were characterized for the same composition, which we assign to Na_8_B_4_Si_42_, allowing for a direct comparison of structural features. Nonetheless, the finding of a clathrate VIII was a surprise because our experiments aimed to complete the clathrate I borosilicides *M*_8–*x*_B_*y*_Si_46–*y*_ (*M* = K, Rb, Cs) with *M* = Na. In this series, only the representative K_7_B_7_Si_39_, which is remarkably stable against oxidizing environments,^16,17^ was prepared at ambient pressure. The clathrate I borosilicides with large cations Rb_8_B_8_Si_38_^18^ and Cs_8_B_8_Si_38_^19^ only form under high-pressure conditions. Consistently, binary clathrate I silicides such as Ba_8_Si_46_^3^ or Cs_8–*x*_Si_46_^20^ are high-pressure phases as well. This finding can be explained by the flexibility of the clathrate network under pressure and the high coordination number achieved for the *M* atoms (pressure-coordination rule^21^). For the smaller alkali metal Na, preparation at ambient pressure conditions did not result in a clathrate phase either. For experiments under high-pressure conditions, a 5:2 mixture of finely ground NaSi^22^ and activated amorphous boron^23^ was filled into BN crucibles. After a reaction time of 45 min at *p* = 5 GPa and *T* = 1200(100) K, XRPD data revealed the formation of a clathrate I phase and a clathrate VIII phase in similar amounts. When the pressure was raised to 6 GPa, the clathrate I phase was no longer observed in XRPD, and clathrate VIII became the majority phase, evidencing that clathrate VIII Na_8–*x*_B_*y*_Si_46–*y*_ is a high-pressure form. In addition, a hexagonal minority phase with an estimated composition of Na_2_Si_2_B_6_^24^ is formed. The highest yield of clathrate VIII was obtained after a reaction time of only 10 min (Figure 2). Without adding boron, a clathrate VIII phase was not observed in the system.

**Figure 1:**
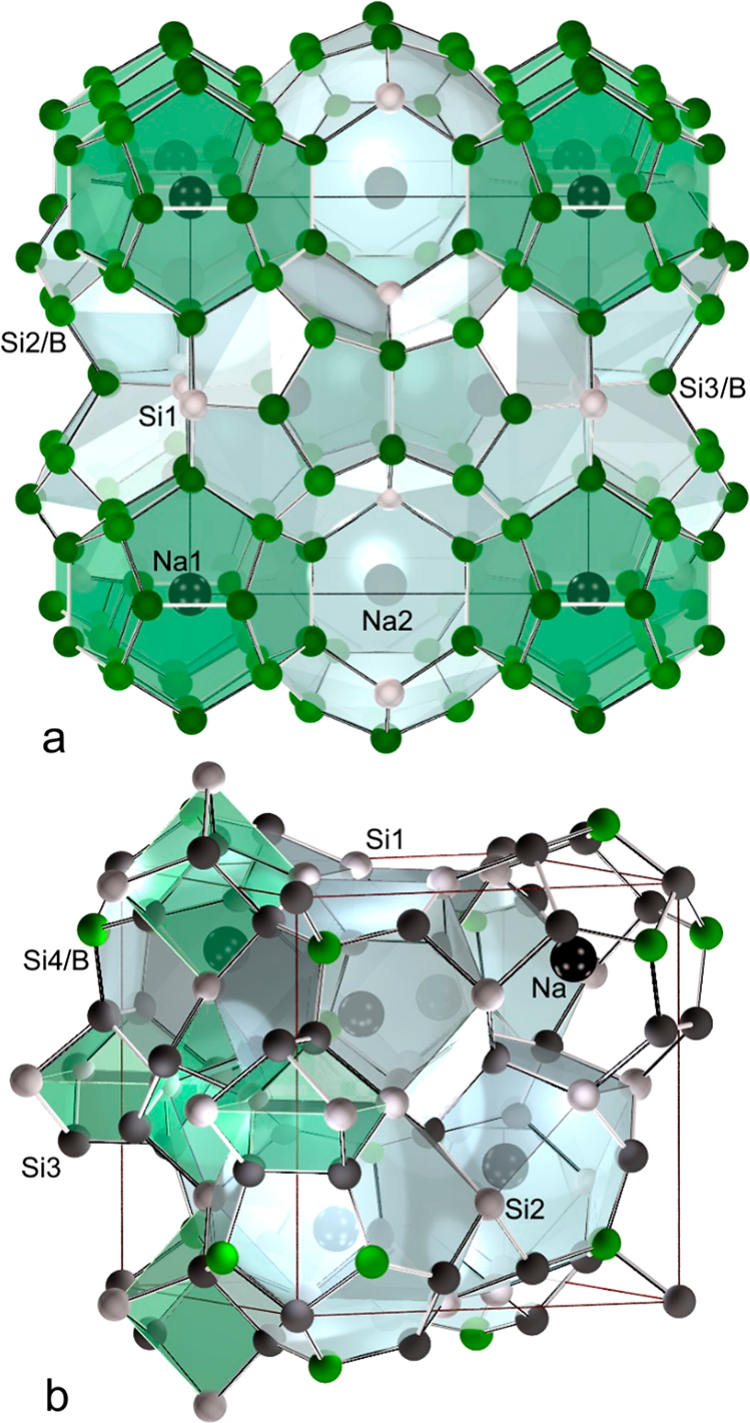
Modifications of Na_8_B_4_Si_42_. (a) Clathrate I structure with 24- (gray) and 20-atom (green) cages (b) Clathrate VIII type structure with Na@*E*_20_ polyhedra (gray) and empty E_8_ realgar voids (green) (E = Si, B). The E–E bonds are indicated in gray.

**Figure 2:**
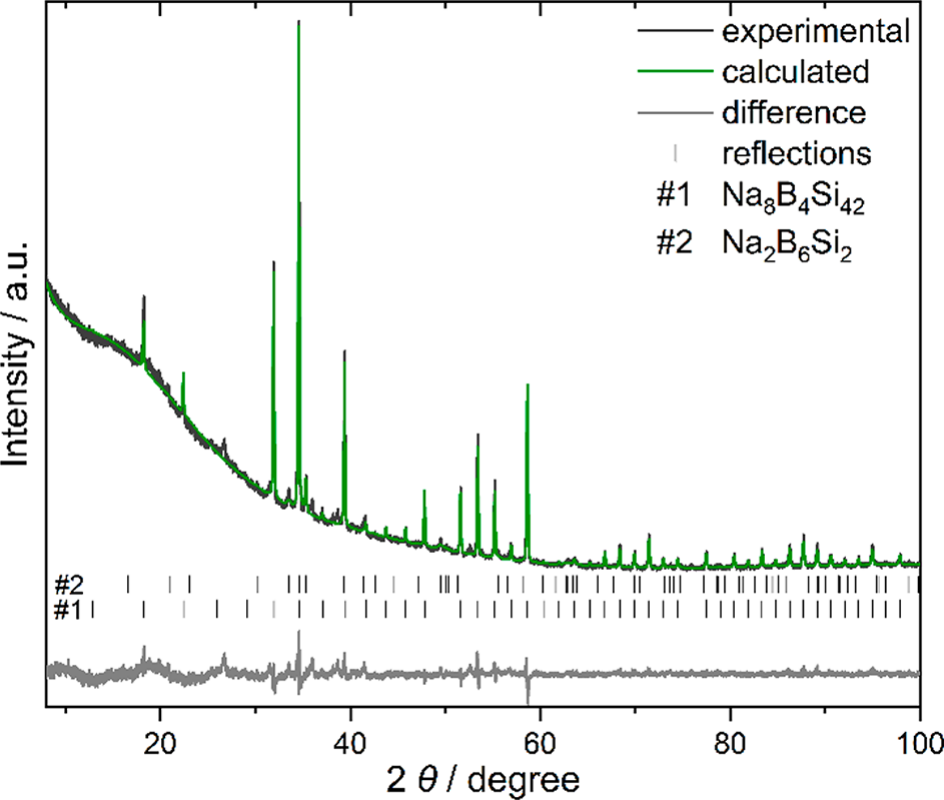
XRPD of a sample obtained at 6 GPa and 950 °C containing clathrate VIII Na_8_B_4_Si_42_ (82(1) mass %) and Na_2_B_6_Si_2_ (18(1) mass %).

For transmission electron microscopy studies, thin lamellas were prepared using the focused ion beam technique.^25^ The sample was investigated by selected-area electron diffraction tomography (SA-EDT). The structure solution from precession data (922 symmetry-independent reflections) revealed the clathrate VIII structure (see the Supporting Information). A qualitative SEM/EDXS analysis of the same specimen area confirmed a ternary main phase consisting of Na, B, and Si (Na:Si ≈ 1:4). The final clathrate VIII structure model was determined from single-crystal X-ray diffraction data. The structure refinement in space group *I*43̅*m* started with a binary model Na_8_Si_46–*x*_ comprising the Wyckoff positions, Si1 (12*d*), Si2 (2*a*), Si3 (24*g*), Si4 (8*c*), and Na (8*c*) centering the 20 + 3-atom cage (Figures 3a and 4). After the atomic positions, the site occupancies, and the atomic displacement parameters (ADP) were refined. Only position Si4 showed a mixed occupancy described by 4 Si and 4 B atoms. Because the bond distances *d*(Si–B) ≈ 2.0 Å and *d*(Si–Si) ≈ 2.3 Å differ, structural disorder occurs. The refined distance values *d*(Si4/B–Si3) = 2.15 Å and *d*(Si4/B–Si2) = 2.26 Å thus represent the mean distances of different local environments. The ADPs of the Si3 atoms feature a cigar-shaped anisotropy with the long axis of the ellipsoid directed toward the center of the empty 8-atom cage (Figure 3e). The anisotropy can be alternatively described by closely adjacent split positions Si31 and Si32 (see Supporting Information). Position Si3 is fully occupied within experimental error, but because of the high site multiplicity, even a minute boron content would significantly change the composition. Moreover, the ADPs of Si2 are slightly enlarged because Si2 is surrounded by 4 Si4/B positions (Figure 4). The presence of vacancies at Si4 instead of boron atoms cannot be ruled out from occupancy refinement. However, such Zintl defects typically cause more pronounced ADP than observed for Si2 and Si3.^26^ Assuming that boron atoms only occupy position Si4, the refinement resulted in the composition Na_8_B_4.2(1)_Si_41.8(1)_ and the residual *R* = 0.03.

**Figure 3:**
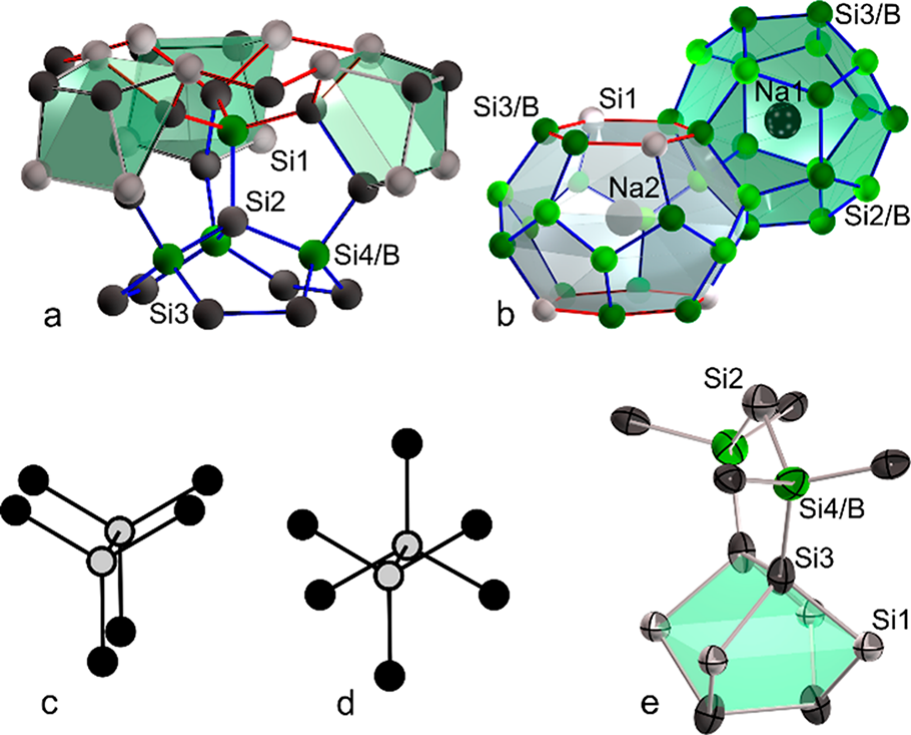
(top) Six- (red) and five-membered rings (blue) in clathrates I and VIII. (a) Alternative representation of the clathrate VIII structure with empty realgar cages (green). (b) Condensed 24- (gray) and 20-atom (green) cages in the clathrate I structure. (bottom) Eclipsed (c) and staggered (d) conformation. (e) Anisotropic displacement ellipsoids of clathrate VIII Na_8_B_4_Si_42_.

**Figure 4:**
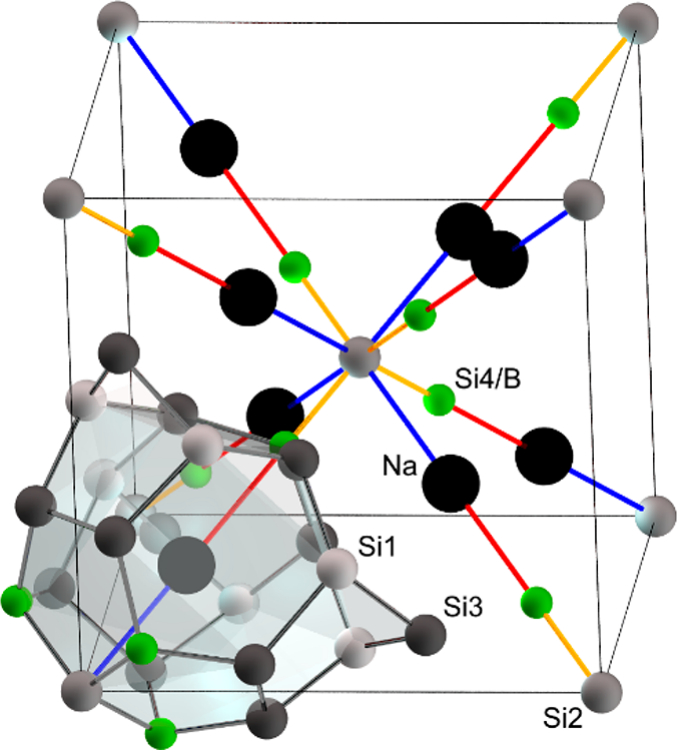
Clathrate VIII arrangement with emphasis on the space diagonal and distances *d*_Na–Si4/B_ (red), *d*_Na–Si2_ (blue), and *d*_Si2–Si4/B_ (orange) along ⟨111⟩. Other atoms are omitted for clarity.

The preference of B atoms for the Si4 position can be rationalized by the close contact with the adjacent Na atoms. Both Na and Si4 occupy individual 8*c* positions (*xxx*) on the space diagonal, allowing for a scaling toward the optimal distance under a strong electrostatic interaction. In the 23-atom cage, Na is surrounded by four Si4 positions. Na always approaches the one located on the same space diagonal. Therefore, the Na positions are off-centered. With the short distance *d*_Na–Si4/B_ = 2.988(5) Å on the space diagonal, the structure adapts to the small lattice parameter (Table 1, Figure 4). This distance is distinctly smaller than the opposite distance *d*_Na–Si2_ = 3.171(3) Å but similar to *d*_Na–Si_ = 2.876 Å in Na_4_Si_4_.^22^

**Table 1:** Selected Interatomic Distances in Clathrate I and VIII Na_8_B_4.1_Si_41.9_

clathrate I		clathrate VIII	
atoms	*d* [Å]	atoms	*d* [Å]
Na1–8 Si2/B	3.216(3)	Na1–1 Si2	3.171(3)
Na1–12 Si3/B	3.259(2)	Na1–3 Si3	3.127(3)
Na2–4 Si1	3.5274(4)	Na1–6 Si3	3.221(2)
Na2–8 Si2/B	3.696(2)	Na1–1 Si4/B	2.988(5)
Na2–8 Si3/B	3.382(2)		
Si1–Si3/B	2.351(2)		
Si2/B–Si2/B	2.208(5)	Si1–Si3	2.3530(8)
Si2/B–Si3/B	2.300(2)	Si2–Si4/B	2.257(3)
Si3/B–Si1	2.351(2)	Si3–Si3	2.343(3)
Si3/B–Si3/B	2.371(4)	Si3–Si4/B	2.151(2)

Interestingly, the off-centering of the cage atom is a feature observed for all clathrate VIII type structures, even for the optimized hypothetical binary Ba_8_Si_46_.^27^ The magnitude of the off-center displacement can be attributed to the extent of the respective ionic interaction. Depending on the synthesis pressure (5–8 GPa), the lattice parameter of clathrate VIII Na_8_B_*x*_Si_46–*x*_ varies from *a* = 9.7579(6) to 9.6750(4) Å. In the type I clathrate K_8–*y*_B_*x*_Si_46–*x*_,^17^ the substitution of Si by B causes a reduction of the lattice parameter of Δ*a* ≈ 0.04 Å/atom. Therefore, with *a* = 9.7187(2) Å for Na_8_B_4.1_Si_41.9_, a composition range of Na_8_B_3_Si_43_–Na_8_B_5_Si_41_ can be estimated. The electron balance (Na^+^)_8_[(4*b*)(B^–^)_4.1_][(4*b*)(Si^0^)_38_]·3.9 *e*^–^ reveals ≈4 excess electrons per formula unit. Consequently, the clathrate VIII phase significantly deviates from an electron-precise composition. We assume the cages become too small to accommodate the Na atoms at higher boron contents.

Among the series of clathrate VIII crystals tested for data collection, one revealed the clathrate I type of structure, although the phase is not visible in the XRPD pattern (Figure 2). We assume that the slow cooling rate of the die may allow for a partial phase transformation on cooling. Crystals of the byproduct Na_2_Si_2_B_6_ were not identified. For the clathrate I borosilicide, the refinement strategy has been described in detail.^8^ The crystal structure in *Pm*3̅*n* features three framework positions, Si1 (6*c*), Si2 (16*i*), Si3 (24*k*), and two Na positions at 2*a* (Na1) and 6*d* (Na2). Boron occupancy is found for position Si2 and, to a small extent, for Si3, whereas Si and Na fully occupy Si1, Na1, and Na2. Boron atoms at the Si2 position are a peculiarity of clathrate I borosilicides, in which the boron atoms prefer positions of the small 20-atom cage. Typically, foreign atoms preferably occupy the six rings of the 24-atom cages (Figure 3b). The ADPs of the Na2 atoms in the larger 24-atom cage show a disc-shaped electron density. ADPs of Si2 are elongated in the direction of the Si2–Si2 bond. Because of mixed occupancy with B and Si, the Si2–Si2 distance represents the superposition of a Si–Si and a B–Si bond (Table 1). Because of the low scattering power of the crystal, split positions for Si2^17^ are not resolved. The refinement results in a residual value *R*_F_ = 0.05 and the composition Na_8_B_4.1(7)_Si_41.9(7)_. Consequently, the clathrate I phase is the only exception in the clathrate I series *M*_8–*x*_B_*y*_Si_46–*y*_ (M = Na, K, Rb, Cs) that deviates significantly from Zintl’s rule. We attribute the low boron content of the clathrate I crystal to the depletion of boron in the reaction mixture because of the formation of Na_2_Si_2_B_6_.

The lattice parameter *a* = 9.7187(2) Å of clathrate VIII Na_8_B_4_Si_42_ is distinctly smaller than *a* = 9.977(2) Å for the clathrate I modification (Table 2), setting a new benchmark for the smallest lattice parameter observed for clathrate phases. (e.g., Na_8_Si_46_, *a* = 10.19(2) Å^28^). The higher density of clathrate VIII is caused by the bond conformation within the four-bonded framework. In the densely packed diamond-type structure of α-Si, all (Si_3_Si)_2_ units have the energetically favored staggered configuration, while the units are eclipsed in clathrate I and clathrate II (Figure 3c,d). The eclipsed conformation leads to the open framework structure with polyhedral cavities and flat five- and six-membered rings (Figure 3b). However, in the clathrate VIII structure, 52% of the bonds feature a staggered arrangement so that the structure is closer to the diamond type. The presence of both corrugated six and flat five-membered rings (Figure 3a) leads to a higher dispersion of bond angles in the framework of clathrate VIII (average *x̅* = 109.3°, standard deviation σ = 9.5°) compared to clathrate I (*x̅* = 109.4°, σ = 4.8°).

**Table 2:** Lattice Parameter and Average Distances *d̅* in Clathrate Borosilicides

composition	*a* [Å]	*d̅* [Å]	ref
Na_8_B_4.2(1)_Si_41.8(1)_ (Cl–VIII)	9.7187(2)	2.302	this work
Na_8_B_4.1(7)_Si_41.9(7)_ (Cl–I)	9.977(2)	2.320	this work
K_7.12(4)_B_7.1(6)_S_i38.9(6)_	9.9393(2)	2.319	(8)
K_7.85(2)_B_7.8(1)_Si_38.2(1)_	9.9050(2)	2.301	(8)
Rb_8_B_7.9(1)_Si_38.1(1)_	9.9583(1)	2.315	(9)
Cs_8.0(1)_B_8.0(1)_Si_38.0(1)_	10.0312(3)	2.332	(10)

The electric transport behavior has not been determined as all syntheses resulted in polycrystalline products with impurity phases. Nevertheless, metallic behavior and low thermoelectric efficiency are expected because both clathrates are not electron balanced. For the clathrate VIII sample with composition Na_8_B_4_Si_42_, no superconducting transition is observed down to 1.9 K (see Supporting Information).

In conclusion, two clathrates occur as high-pressure phases in the ternary system of sodium, boron, and silicon and are structurally characterized for the composition Na_8_B_4_Si_42_. The clathrate I modification completes the series *M*_8–*x*_B_*y*_Si_46–*y*_ (M = K, Rb, and Cs). The clathrate VIII modification is the first borosilicide crystallizing in this rarely observed structure type. The high content of bonds with staggered conformation leads to a more compact packing than in clathrate I, resulting in the smallest lattice parameter for a silicon clathrate. The formation of clathrate VIII at the cost of clathrate I upon pressure enhancement opens the perspective for preparing new members of this rare, nevertheless attractive, structure type.

## Supporting Information Available

Synthesis, materials, and characterization details, and additional results including crystallographic data (PDF)
