# Supplementary material for: A Borosilicide with Clathrate VIII Structure
Source: J Am Chem Soc. 2022 Jul 25;144(30):13456–60. doi: 10.1021/jacs.2c04745 (PMC9377393; doi:10.1021/jacs.2c04745)
Supplement: Supplementary file 1 — ja2c04745_si_001.pdf [file ja2c04745_si_001.pdf]

Supporting information:

A borosilicide with clathrate VIII structure

Julia-Maria Hübner[1,2]*, Wilder Carrillo-Cabrera[2], Primoz Kozelj[2], Yurii Prots[2], Michael Baitinger[2], Ulrich Schwarz[2], Walter Jung[2]

[1]Centre for Analysis and Synthesis, Department of Chemistry, Naturvetarvägen 14, 221 00 Lund, Sweden
[2]Max-Planck-Institute for Chemical Physics for Solids, 01187 Dresden, Germany.

1. Preparation
2. Crystal Structure of the clathrate VIII phase from electron diffraction data
3. Crystallographic data of the clathrate VIII phase from single-crystal X-ray diffraction data
4. Crystallographic data of the clathrate I phase from single-crystal X-ray diffraction data
5. Magnetic susceptibility

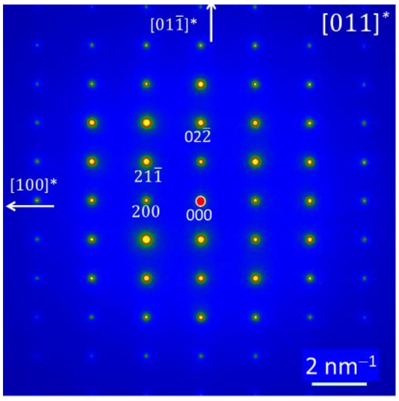

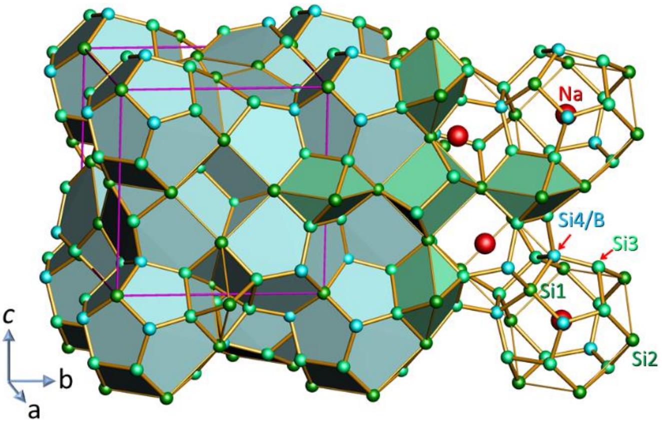

1. Preparation

Sample handling was conducted in an argon-filled glove box except for the high-pressure synthesis and the washing procedure of the product. The precursor compound Na_4Si_4 was synthesized from sodium (Chempur, 99.95%) and silicon (Chempur, 99.9999%) in a closed tantalum tube by annealing at 750 °C for 7 h, followed by slow cooling to room temperature within 8 h. Amorphous boron (Alfa Aesar) was purified and activated in a streaming hydrogen plasma [1]. The educt mixtures with molar ratio Na:B:Si = 5:2:5 were thoroughly ground in agate mortars. The mixture was filled into a boron nitride crucible of 3 mm diameter and placed in MgO octahedra with an edge length of 18 mm. The high-pressure, high-temperature syntheses were performed in a multi-anvil press comprising a Walker-type module [2]. Pressure and temperature were calibrated before the experiments by recording the resistance changes of bismuth and by thermocouple-calibrated runs, respectively. To prepare $Na_8B_4Si_{42}$, a pressure of $p = 6 \pm 1$ GPa was applied at room temperature. After heating to $T = 950 \pm 100$ °C within 15 min and annealing for 10 min, the samples were quenched under load. The reaction products were washed with ethanol and deionized water to remove traces of Na_4Si_4, followed by washing with ethanol and acetone and drying at room temperature.

2. Crystal structure of the clathrate VIII phase from electron diffraction data

The clathrate VIII structure was first identified by combining conventional transmission electron microscopy, selected area electron diffraction (SAED), and manual selected area electron precession diffraction tomography (SA-PEDT). The investigations were performed on an FEI Tecnai F30-G2 super-twin microscope operating at 300 kV. The microscope was equipped with a CCD camera (GATAN Inc.) and a standard double-tilt holder (GATAN Inc.) with a tilting range of ± 46° of the holder axis and ± 30° perpendicular to it. The SAED mode was used for tomography data collection.

Precession electron diffraction (PED) was performed using a DigiStar P1000 device (Nanomegas). The images were stored in dm3 file format, primarily processed and analyzed with Digital Micrograph (version 3.21.1374.0; GATAN Inc.).

A specimen suitable for the TEM investigations was prepared with the focused ion beam technique (FIB) using a Quanta 200 3D ion/electron dual-beam device (FEI, Eindhoven). The device was equipped with an omniprobe micro-manipulator (W needle) and could be used both as a scanning electron microscope (SEM) and a scanning ion microscope (SIM). SEM images revealed, besides smaller grains, crystalline grains with a cubic habit which were used for the subsequent investigations (Fig. 1a).

Prior to the sample cut, protecting Pt layers (24 µm long, 2 µm thickness, 2 µm high) were deposited on the selected part (parallel and perpendicular to the *c* axis of microcrystals arrays, Fig. 1a) using an acceleration voltage of 30 kV and a current of 0.1 nA. Each cross-section (2 µm thickness) was then prepared with a Ga-ion beam with an acceleration voltage of 30 kV and a 1–0.5 nA current. The manufactured cut was transferred onto a copper Omniprobe TEM holder using the in-situ lift-out technique [3]. Subsequently, the cross-section was thinned to a thickness of about 60 nm applying an acceleration voltage of 30 kV and currents of 0.5–0.01 nA of the Ga-ion beam.

The lamella contained a silicon-rich phase, later revealed as a clathrate VIII phase (bright areas in Fig. 1b) and a boron-rich phase (dark areas) with approximate composition '$Na_2B_6Si_2$'. The lamella (red area, Fig. 1b) was further investigated by transmission electron microscopy.

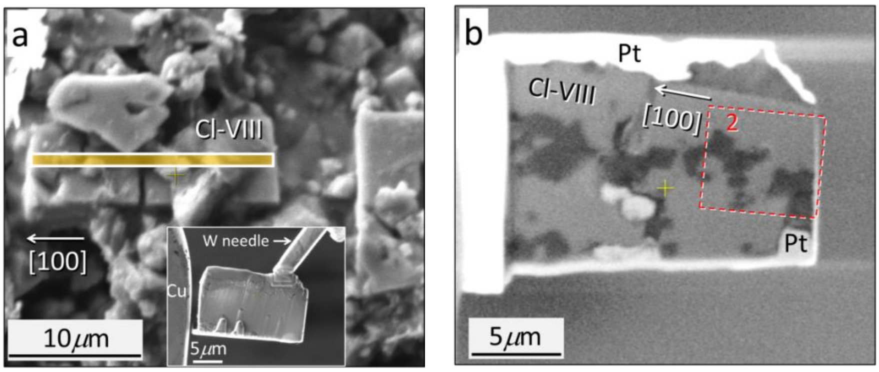

Figure 1. (a) SEM image of crystals with cubic habitus. The area selected for extraction by the FIB technique is marked yellow. The inserted SIM image displays the FIB cross-section after *lift-out* with a W needle before soldering to a Cu TEM holder and further thinning. (b) Thinned FIB lamella (SEM image, back-scattering contrast). Dark gray areas represent the hexagonal boron-rich phase $Na_2B_6Si_2$; the brighter matrix areas belong to the silicon-rich clathrate VIII phase. Some voids were also filled by the protecting Pt-layer (white regions) during the FIB experiment. The area labeled by number 2 (red square) is shown as a TEM image in Fig. 2.

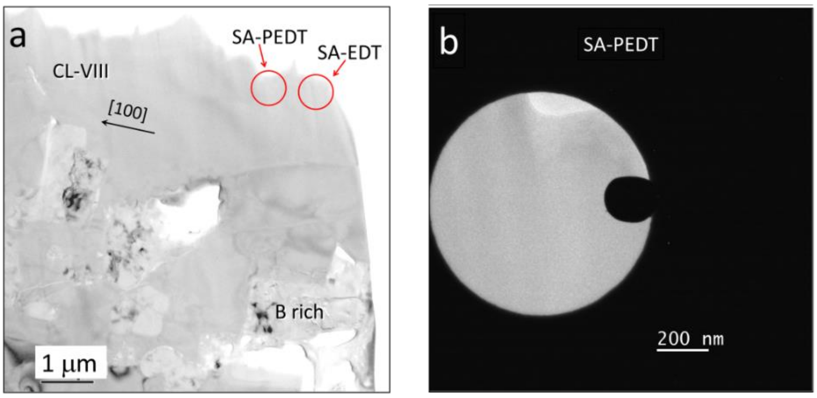

Figure 2. (a) TEM image of the region marked by the red square in Fig. 1b. In turn, the red circles mark the areas selected for the SA-PEDT electron diffraction tomography experiments. (b) Aperture (ca. 850 nm) with the selected area (SA) of the clathrate VIII phase chosen for the 3D-PED data acquisition using electron diffraction tomography (SA-PEDT). The shadow of the beam-stopper tip is also visible. SAED experiments revealed the crystal symmetry and the lattice parameter. The reflection conditions indicate an *I*-centred cubic lattice ($a \approx 9.98$ Å) in agreement with space group *I*-43*m* of the clathrate VIII type (Fig. 3).

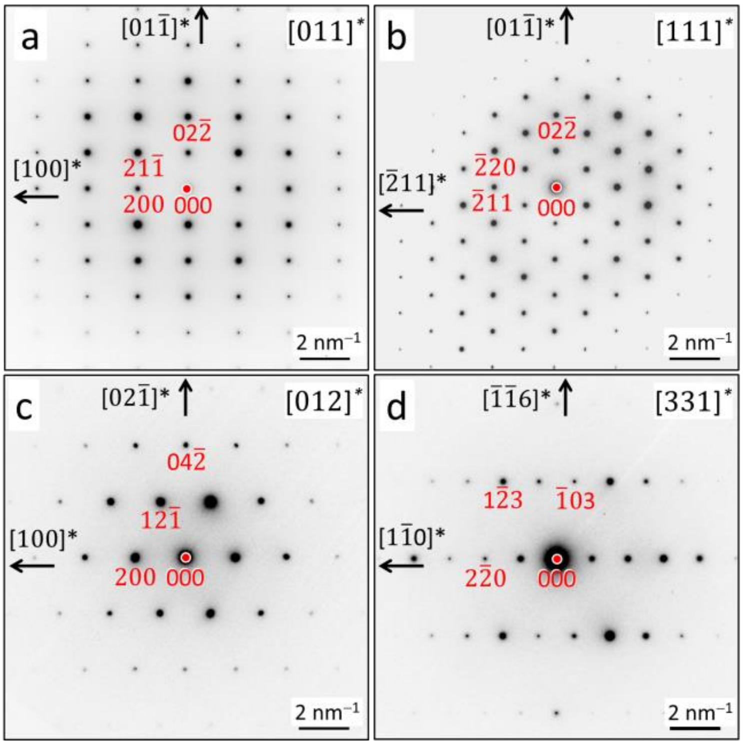

Figure 3. SAED images for the clathrate VIII phase: (a) Along [001]* zone axis, (b) along [111]* zone axis, (c) along [012]* zone axis and (d) along [331]* zone axis. The observed *hkl* reflection conditions are in agreement with those for the spaced group $I\bar{4}3m$, namely: *hkl*, *h+k+l* = 2*n*; *0kl*, *k+l* = 2*n*; *hhl*, *l* = 2*n*; *h00*, *l* = 2*n*.

An SA-EDT electron diffraction tomography experiment (camera length 560 mm) revealed the clathrate-VIII type of structure. However, strong dynamic multi-beam effects biased the recorded dataset of 447 measured and 212 symmetry-independent reflections so that the structure refinement converged to a high R_{gt} value of 0.45. To reduce the multi-beam dynamical effects, a SA-PEDT experiment was performed with a precession angle of the electron beam of 1.26°. Intensity data were collected in a tilt sequence from -43° to +43° (step-width of 1°). The 2D-PED data (87 images in dm3 file format) were converted to the TIF format to be indexed and integrated with the PETS software package (version 2.0) [4]. Indexing revealed an *I*-centred cubic unit-cell ($a \approx 9.9$ Å). The electron beam precession and the smaller camera length (300 mm) resulted in a larger data set. 7589 reflections were measured, yielding 922 symmetry-independent reflections stored in standard hkl file format. The 3D-PED diffraction volume is shown in Fig. 4 projected along *a**, *b**, and *c** axes.

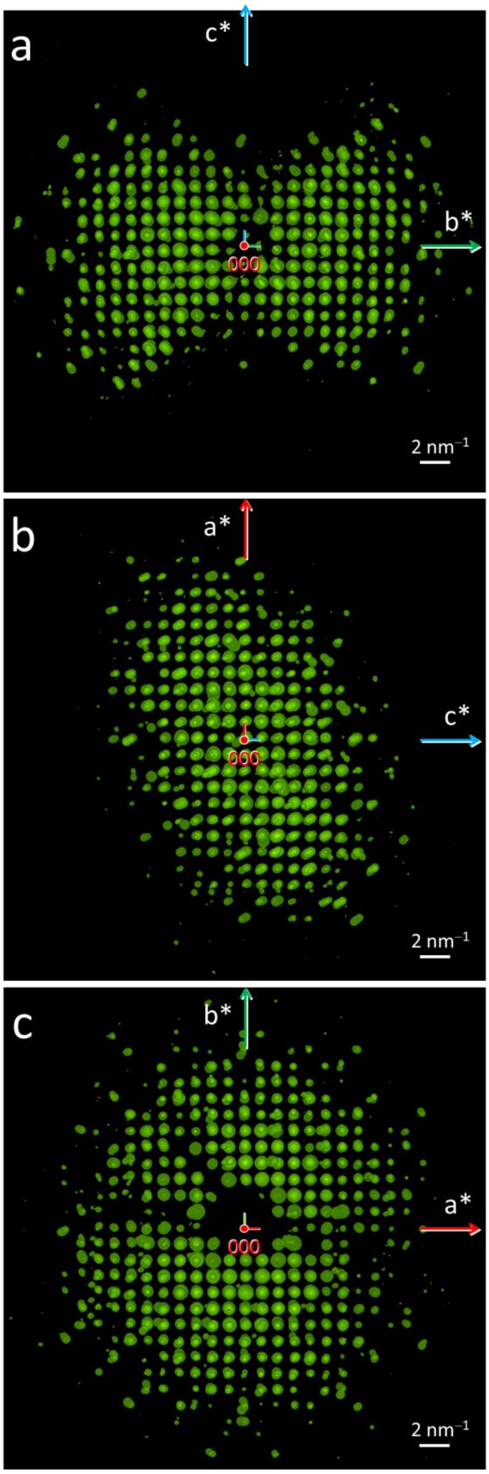

Figure 4. Projections of the 3D-PED diffraction volume of the clathrate VIII phase. The projections are (a) along *a** axis, (b) along *b** axis, and (c) along *c** axis. The 3D-PED diffraction volume (reciprocal-space volume) was reconstructed from 87 2D-SA-PED images (selected-area electron diffraction mode) obtained by a stepwise tilt in the range –43° to 43° (tilt step 1°). The acquisition tilt axis coincides with the TEM goniometer axis (α angle tilting axis). Images and the 3D-PED volume reconstruction were made using the PETS 2.0 software [4].

Crystal structure refinement (Jana2006 software [5]) started with the atomic positions of α-$Eu_8Ga_{16}Ge_{30}$ [6]. Unlike the XRPD study, Boron atoms can be assigned to two crystallographic sites, Si3 and Si4, resulting in the composition $Na_8B_{8(1)}Si_{38}$ (X-ray: $Na_8B_4Si_{42}$). However, the high residue of R_g = 0.20 (816 reflections with $I > 2\cdot\sigma(I)$) caused by multi-beam dynamical effects may bias the structure model. The crystallographic data are listed in Table 1, and the atomic coordinates and displacement parameters are listed in Table 2. Please note that the cif file is based on more accurate single-crystal data (Table 3,4).

Table 1. Crystallographic data of a clathrate VIII single-crystal investigated with an SA-PEDT experiment (293 K).

Formula; Z	'$Na_8B_8Si_{38}$'; 1
Mole mass	1337.7 $g\cdot mol^{-1}$
Space group	$I\bar{4}3m$ (no. 217)
Lattice parameter*	$a \approx$ 9.9 Å
Pearson symbol	*cI*54
Calculated density	2.444 g/cm^3
Data collection	Tecnai F30-G2 super-twin electron microscope (FEI)
Radiation	Electrons; λ = 0.0197 Å
2θ range	0.22° - 2.38°
Reflections (total/independent)	7589 / 922
$I > 2\ \sigma(I)$	816
Absorption/Extinction	Uncorrected data
Analyzing software	PETS [4], Jana2006 [5]
Refined parameters	18
Largest diff. peak, hole	+0.33, –1.14 $e\cdot Å^{-3}$
R_{gt} (electrons)	0.204
Goodness of fit on F^2	7.82

*lattice parameters from ED are only a rough approximation.

Table 2. Atomic coordinates and isotropic displacement parameters of a clathrate VIII single-crystal derived from the SA-PEDT experiment.

Atom	site	*x/a*	*y/b*	*z/c*	$U_{eq}/Å^2$
Na	8*c*	0.1914(4)	*x*	*x*	0.0362(9)
Si1	12*d*	1/4	1/2	0	0.0180(6)
Si2	2*a*	0	0	0	0.0413(16)
Si/B3 [a)]	24*g*	0.4161(2)	*x*	0.1492(3)	0.0172(5)
Si/B4 [b)]	8*c*	0.3659(3)	*x*	*x*	0.0201(9)

a) Occupancy [Si/B3] = 0.24(5)B + 0.76Si. [b)] Occupancy [Si/B4] = 0.27(9)B + 0.73Si.

3. Crystallographic data of the clathrate VIII phase from single-crystal X-ray diffraction data

Structure refinement with Shelx software [7] against F^2 resulted in the composition $Na_8B_{4.2(1)}Si_{41.8(1)}$ and a residual value of $R(F^2)$ = 0.03 (Table 1). Refinement with WINCSD software [8] against F resulted in the composition $Na_8B_{4.2(1)}Si_{41.8(1)}$. The real value cannot be decided based on our data, and we estimate the accuracy of the composition to $Na_8B_{4(1))}Si_{42(1)}$. Tables 3-5 present the results of the structure refinement with Shelx, used in the main text. Alternatively, Table 6 shows a split atom model refined with WINCSD.

Table 3. Crystallographic data of a clathrate VIII single-crystal investigated with X-ray diffraction (293 K). Further details on the crystal structure investigations can be obtained from the FachinformationszentrumKarlsruhe, 76344 Eggenstein-Leopoldshafen, Germany (email: crysdata@fiz-karlsruhe.de, http:///www.fiz-karlsruhe.de/request_for_deposited_data.html) on quoting the depository numbers CSD-2169575.

Formula; *Z*	$Na_8B_{4.2(1)}Si_{41.8(1)}$; 1
Mole mass	1403.3 g·mol^{-1}
Space group	$I\bar{4}3m$ (no. 217)
Pearson symbol	*cI*54
Lattice parameter	a = 9.7187(2) Å
Volume	923.8(3) Å^3
Diffractometer	Rigaku Xcalibur EOS S2, CCD detector, graphite monochromator,
Radiation	Mo Kα radiation, λ = 0.71073 Å
Reflections collected	11027
independent within I > 3σ(I)	344
Measurement range	$-14 \le h \le 14$, $-14 \le k \le 14$, $-14 \le l \le 14$
Residuals and GOF	$R(F^2)$ = 0.0302, $wR(F^2)$ = 0.0633, GOF = 1.137
Analyzing software	Shelx [8], WinCSD [9]

Table 4. Position and displacement parameters for clathrate-VIII $Na_8B_{4.2}Si_{41.8}$

Atom	Site	*a* / x	*b* / y	*c* / z	U_{eq}*	Occ.
Na1	8*c*	0.1884(2)	*x*	*x*	0.0233(7)	1
Si1	12*d*	0.25	0.5	0	0.0124(3)	1
Si2	2*a*	0	0	0	0.0186(8)	1
Si3	24*g*	0.41475(9)	*x*	0.1556(1)	0.0153(3)	1
Si4/B	8*c*	0.3659(2)	*x*	*x*	0.017(1)	0.48(1) Si<br>0.52(1) B

*U_{eq} is defined as one third of the trace of the orthogonalized U_{ij} tensor. $U_{ij} = 2\pi^2[(ha^*)^2 U_{11} + ... + 2hka^*b^*U_{12}]$.

Na1: $U_{11} = U_{22} = U_{33}$ = 0.0233(7), $U_{12} = U_{13} = U_{23}$ = -0.0047(7);
Si1: U_{11} = 0.0137(7), $U_{22} = U_{33}$ = 0.0118(4), $U_{12} = U_{13} = U_{23}$ = 0;
Si2: $U_{11} = U_{22} = U_{33}$ = 0.0186(8), $U_{12} = U_{13} = U_{23}$ = 0;
Si3: $U_{11} = U_{22}$ = 0.0121(3), U_{33} = 0.0217(6), U_{12} = 0.0016(4), $U_{13} = U_{23}$ = -0.0021(3);
Si4/B: $U_{11} = U_{22} = U_{33}$ = 0.017(1), $U_{12} = U_{13} = U_{23}$ = -0.0025(7)

Table 5. Selected interatomic distances in clathrate-VIII $Na_8B_{4.2}Si_{41.8}$.

Atom	Distance / Å	Atom	Distance / Å	Number of Si-Si bonds
Na1 – 1 Si2	3.171(3)	Si1 – 4 Si3[e]	2.3530(8)	48
Na1 – 3 Si3	3.127(3)	Si2 – 4 Si4/B[s]	2.257(3)	8
Na1 – 6 Si3	3.221(2)	Si3 – 2 Si1[e]	2.3530(8)	48
Na1 – 1 Si4/B	2.988(5)	Si3 – 1 Si3[s]	2.343(3)	24
Na1 – 3 Si4/B	3.221(2)	Si3 – 1 Si4/B[s]	2.151(2)	24
		Si4/B – 1 Si2[s]	2.257(3)	8
		Si4/B – 3 Si3[s]	2.151(2)	24

[e] eclipsed, [s] staggered

Table 6. Position and displacement parameters for clathrate-VIII $Na_8B_{4.7(3)}Si_{41.3(3)}$ using a split atom model.

Atom	Site	*a* / x	*b* / y	*c* / z	B_{eq}*	Occ.
Na1	8*c*	0.1879(4)	*x*	*x*	2.27(7)	1
Si1	12*d*	0.25	0.5	0	1.15(5)	1
Si2	2*a*	0	0	0	1.32(7)	1
Si31	24*g*	0.4143(7)	*x*	0.1655(8	1.20(10)	0.5(2)
Si32		0.4151(7)	*x*	0.1486(7)	1.24(12)	0.5(2)
Si4/B	8*c*	0.3653(4)	*x*	*x*	0.017(1)	0.41(4) Si<br>0.59(4) B

*$B_{(eq)} = 1/3[B_{11}\, a^{*2}\, a^2 + \ldots 2B_{23}\, b^*\, c^*\, b\, c \cos(\alpha)]$

Na1: $B_{11} = B_{22} = B_{33} = 2.27(12)$, $B_{12} = B_{13} = B_{23} = -0.47(12)$;
Si1: $B_{11} = 1.31(10)$, $B_{22} = B_{33} = 1.06(7)$, $B_{12} = B_{13} = B_{23} = 0$;
Si2: $B_{11} = B_{22} = B_{33} = 1.32(12)$, $B_{12} = B_{13} = B_{23} = 0$;
Si4/B: $B_{11} = B_{22} = B_{33} = 1.2(2)$, $B_{12} = B_{13} = B_{23} = 0.02(12)$

4. Crystallographic data of the clathrate I phase from single-crystal X-ray diffraction data

Table 7. Crystallographic data of a clathrate I single-crystal $Na_8B_{4.1}Si_{41.9}$ investigated with X-ray diffraction (293 K). Further details on the crystal structure investigations can be obtained from the FachinformationszentrumKarlsruhe, 76344 Eggenstein-Leopoldshafen, Germany (email: crysdata@fiz-karlsruhe.de, http:///www.fiz-karlsruhe.de/request_for_deposited_data.html) on quoting the depository numbers CSD- 2169576.

Formula; *Z*	$Na_8B_{4.1(7)}Si_{41.9(7)}$; 1
Mole mass	1405.0 g·mol^{-1}
Space group	*Pm*$\overline{3}$*n (No. 223)*
Pearson symbol	*cP*54
Lattice parameter	*a* = 9.977(1) Å
Volume	993.1(4) Å^3
Diffractometer	Rigaku Xcalibur EOS S2, CCD detector, graphite monochromator,
Radiation	Mo Kα radiation, λ = 0.71073 Å
Reflections collected	11783
independent within I > 3σ(I)	176
Measurement range	$-11 \le h \le 11$, $-11 \le k \le 11$, $-11 \le l \le 11$
Residuals and GOF	$R(F^2)$ = 0.0529, $wR(F^2)$ = 0.0945, GOF = 1.518
Analyzing software	Shelx [8]

Table 8. Position and displacement parameters for clathrate I $Na_8B_{4.1}Si_{41.9}$.

Atom	Site	*a* / x	*b* / y	*c* / z	U_{eq}*	Occ.
Na1	2*a*	0	0	0	0.025(2)	1.0
Na2	6*d*	0.25	0.5	0	0.046(2)	1.0
Si1	6*c*	0.25	0	0.5	0.016(1)	1.0
Si2/B	16*i*	0.1861(2)	*x*	*x*	0.0185(8)	0.826(7) Si<br>0.174(7) B
Si3/B	24*k*	0	0.3042(2)	0.1188(2)	0.0186(7)	0.947(5) Si<br>0.053(5) B

*U_{eq} is defined as one third of the trace of the orthogonalized U_{ij} tensor. $U_{ij} = 2\pi^2[(ha^*)^2 U_{11} + ... + 2hka^*b^*U_{12}]$.

Na1: $U_{11} = U_{22} = U_{33} = 0.025(2)$, $U_{12} = U_{13} = U_{23} = 0$

Na2: $U_{11} = 0.034(4)$, $U_{22} = U_{33} = 0.052(3)$, $U_{12} = U_{13} = U_{23} = 0$

Si1: $U_{11} = 0.017(2)$, $U_{22} = U_{33} = 0.016(1)$, $U_{12} = U_{13} = U_{23} = 0$

Si2/B: $U_{11} = U_{22} = U_{33} = 0.0185(8)$, $U_{12} = U_{13} = U_{23} = 0.023(7)$

Si3/B: $U_{11} = 0.023(1)$, $U_{22} = U_{33} = 0.017(1)$, $U_{23} = -0.0035(9)$, $U_{12} = U_{13} = 0$

Table 9. Selected interatomic distances in clathrate I $Na_8B_{4.1}Si_{41.9}$.

Atom	Distance / Å	Atom	Distance / Å
Na1 – 8 Si2/B	3.216(3)	Si1 – 4 Si3/B	2.351(2)
Na1 – 12 Si3/B	3.259(2)	Si2/B – 1 Si2/B	2.208(5)
Na2 – 4 Si1	3.5274(4)	Si2/B – 3 Si3/B	2.300(2)
Na2 – 8 Si2/B	3.696(2)	Si3/B – 1 Si1	2.351(2)
Na2 – 8 Si3/B	3.382(2)	Si3/B – 1 Si2/B	2.300(2)
		Si3/B – 1 Si3/B	2.371(4)

5. **Magnetic susceptibility**

The magnetic susceptibility was measured using a polycrystalline sample of cylindrical shape on a squid magnetometer (MPMS XL-7, Quantum Design) between 1.8 and 350 K in external fields of 15000 to 7000 Oe.

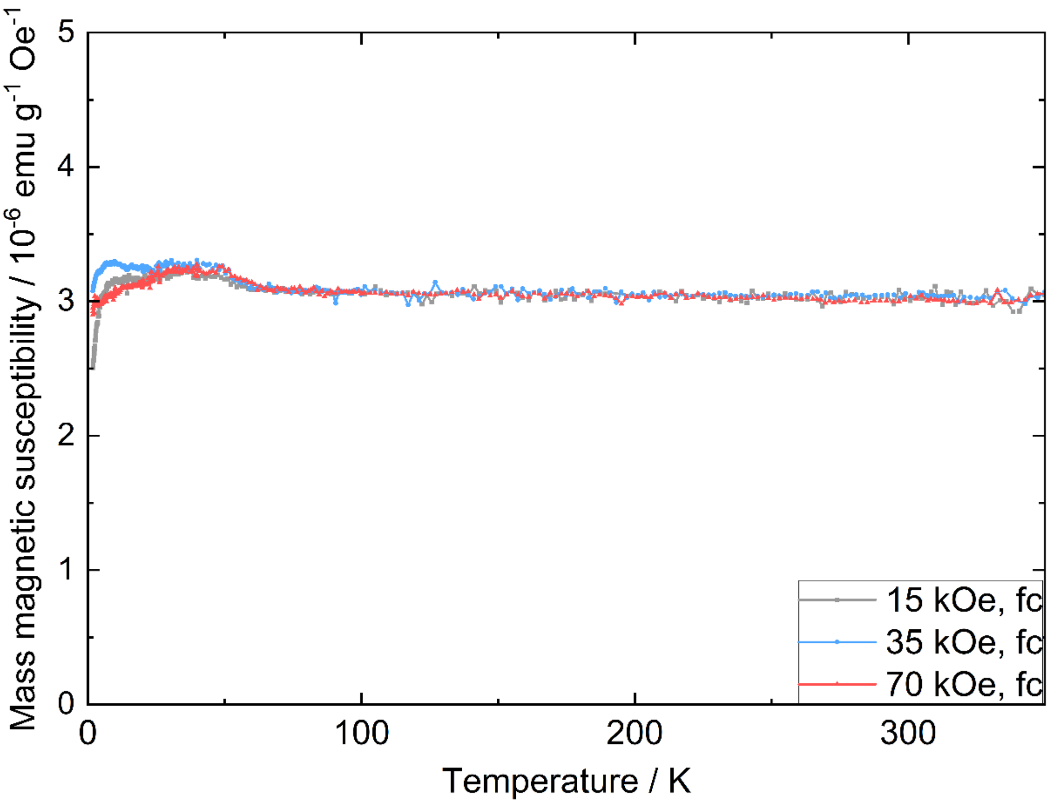

Figure 5. Mass magnetic susceptibility χ of clathrate VIII $Na_8B_{4(1)}Si_{42(1)}$ between 1.8 and 350 K.

Due to the byproduct $Na_2B_6Si_2$ in the sample, the evaluation of absolute values was unfeasible. However, the Pauli paramagnetism of the phase mixture is in line with the expected metallic behavior and superconductivity can be ruled out down to 1.9 K.

6. References

[1] Alekseeva, A.; Kovnir, K.; Chizhov, P.; Baitinger, M.; Grin, Yu. Materials purification by treatment with hydrogen-based plasma. Eur. Pat. EP1893320 B8, **2010**.

[2] Walker, D.; Carpenter, M. A.; Hitch, C. M. *Am. Mineral.* **1990**, 75, 1020– 1028.

[3] Fan, J.; Carrillo-Cabrera, W.; Akselrud, L.; Prots, Y.; Antonyshyn, I.; Chen, L.; Grin, Yu., *Inorg. Chem.* **2013**, 52, 11067–11074.

[4] Palatinus, L.; Brázda, P.; Jelínek, M.; Hrdá, J.; Steciuk, G.; Klementova, M., *Acta Cryst.* **2019**, *B75*, 512–522.

[5] Petricek, V.; Dusek, M.; Palatinus, L., *Z. Kristallogr.*, **2014**, 229, 345−352.

[6] Paschen, S.; Carrillo-Cabrera, W.; Bentien, A.; Tran, V. H.; Baenitz, M.; Grin, Yu.; Steglich F., *Phys. Rev.*, **2001**, B64, 214404.

[7] Sheldrick, G. M. *Acta Cryst.* **2008**, A64, 112-122.

[8] Akselrud L.; Grin, Yu. *J. Appl. Crystallogr.*, **2014**, 47 , 803.
